# Respiratory Syncytial Virus-Specific Antibodies and Atopic Diseases in Children: A 10-Year Follow-Up

**DOI:** 10.3390/pathogens12040546

**Published:** 2023-04-01

**Authors:** Helena Tesari Crnković, Krešo Bendelja, Vlado Drkulec, Romana Gjergja Juraški, Mirjana Turkalj

**Affiliations:** 1 Department of Paediatrics, General County Hospital Požega, Osječka 107, 34000 Požega, Croatia; 2 Faculty of Medicine, J. J. Strossmayer University of Osijek, J. Huttlera 4, 31000 Osijek, Croatia; 3 Center for Research and Knowledge Transfer in Biotechnology, University of Zagreb, Rockefeller Street 10, 10000 Zagreb, Croatia; 4 Neuropaediatric Department, Srebrnjak Children’s Hospital, Srebrnjak 100, 10000 Zagreb, Croatia; 5 Department of Pulmonology and Allergology, Srebrnjak Children’s Hospital, Srebrnjak 100, 10000 Zagreb, Croatia; 6 School of Medicine, Catholic University of Croatia, Ilica 242, 10000 Zagreb, Croatia

**Keywords:** respiratory syncytial virus, children, immunoglobulin G4, immunoglobulin E, wheezing, asthma, atopic dermatitis, allergic rhinitis

## Abstract

Background: Respiratory syncytial virus (RSV) stimulates the production of specific immunoglobulin (Ig) E and IgG4 antibodies as a hallmark of the Th2 immune response. In this paper, we evaluated the occurrence of atopic diseases in 10-year-old children who were positive for RSV-specific IgG antibodies during infancy. Methods: The prospective follow-up of 72 children included a physical examination, an International Study of Asthma and Allergies in Childhood (ISAAC) questionnaire and the determination of RSV-specific antibodies and total and allergen-specific IgE. Results: Children with asthma had their first wheezing episode at a younger age (χ2 8.097, df = 1, *p* = 0.004). RSV-specific IgG4 levels at year one were positively correlated with atopic dermatitis (AD) (tau_b = 0.211, *p* = 0.049) and current AD (tau_b = 0.269, *p* = 0.012); and RSV-specific IgE levels were positively correlated with allergic rhinitis (AR) (tau_b = 0.290, *p* = 0.012) and current AR (tau_b = 0.260, *p* = 0.025). Positive RSV-specific IgE at the age of one increased the chances of asthma occurrence by 5.94 (OR = 5.94, 95% CI = 1.05–33.64; *p* = 0.044) and the chances of AR by more than 15 times (OR = 15.03, 95% CI = 2.08–108.72; *p* = 0.007). A positive family history of atopy increased the chances of asthma occurrence by 5.49 times (OR = 5.49, 95% CI = 1.01–30.07; *p* = 0.049), and a longer duration of exclusive breastfeeding lowered that chance (OR = 0.63, 95% CI = 0.45–0.89; *p* = 0.008). Prenatal smoking increased the chances of AR occurrence by 7.63 times (OR = 7.63, 95% CI = 1.59–36.53; *p* = 0.011). Conclusion: RSV-specific IgE and RSV-specific IgG4 antibodies could be risk markers for the development of atopic diseases in children.

## 1. Introduction

Respiratory syncytial virus (RSV) is the most common viral cause of lower respiratory tract infections in children during the first year of life [1,2]. It is considered that more than 70% of children are infected with RSV in the first year and 90% during the first two years of their life [3]. Reinfections during the winter months are common [4], and they lead to the accumulation of RSV-specific antibodies [5].

Most children infected with RSV are asymptomatic or have mild symptoms, but 2 to 3% are hospitalized due to severe symptoms [6]. The severe forms of infection are bronchiolitis and pneumonia [7]. Particularly severe infections are present in children with risk factors, such as premature neonates or children with compromised cardiorespiratory or immunological systems, for whom preventive therapy with an anti-RSV monoclonal antibody (palivizumab) is conducted [8].

RSV is the cause of recurrent and persistent episodes of wheezing [9,10], and, according to some researchers, it leads to a higher incidence of asthma and asthma persistence [11]. The premise that early postnatal RSV infection is an independent risk factor for asthma is supported by prospective cohort studies [12]. Important factors in the possible development of asthma following RSV infection are the number [13,14,15] and severity [9,16] of infections. Moreover, a younger age at the onset of infection [17,18] is associated with later asthma, although different results have been published [19,20]. An older age at the onset of RSV bronchiolitis is associated with a higher likelihood of asthma in the future, according to the results of Zhou Y. et al. [20].

Some researchers have detected RSV-specific immunoglobulin (Ig) E antibodies in the nasopharynx [21,22,23], and these showed an association with pulmonary function [24]. In the sera of some children infected with RSV, RSV-specific IgE, IgG4 [25,26] and IgG3 antibodies [27] have been found. RSV-specific IgE antibodies are detected in children with asthma [28]. It has been reported that viral infections can initiate the atopic cycle and allergic sensitization [29] or an immediate hypersensitivity reaction and recurrent wheezing [30] via the induction of RSV-specific IgE antibodies. The simultaneous induction of RSV-specific IgG4 antibodies suggests that the production of IgG4-blocking antibodies could control unwanted IgE-induced allergic symptoms [26]. After an acute RSV infection, RSV-specific IgE and IgG4 antibodies are higher in children with wheezing [25].

RSV and rhinovirus (RV) have been identified as risk factors for asthma, especially in the presence of allergic sensitization [13,14]. The “two-hit hypothesis” has been proposed, as there is a synergistic reaction between allergic sensitization and severe lower respiratory tract infection in the development of childhood asthma [31]. The importance of the interaction between viral infection and allergen exposition has been emphasized [32]. The “hit-and-run” hypothesis implies the origin of asthma after viral infection in the appropriate genetic constitution [33]. Atopy is considered an important factor for the development of asthma after RSV infection [20].

## 2. Methods and Materials

This study is a 10-year follow-up conducted as a continuation of our first study in which we monitored the development of atopic diseases (food allergies, atopic dermatitis, and recurrent wheezing) in children up to 2 years of age who had positive RSV-specific IgG antibodies in that time frame [34]. During this research phase, we monitored the children from that specific birth cohort up to the age of 10 to investigate whether there is a connection between the specific subclasses of RSV-specific antibodies sampled during the first 2 years of life and the development of atopic diseases (recurrent wheezing/asthma, atopic dermatitis, allergic rhinitis, and rhinoconjunctivitis) in 10-year-old children.

Our research is an observational prospective cohort study of 127 newborns delivered at the Maternity Department of the General County Hospital Požega, Croatia, from July 2009 to September 2010. At prenatal gynecological check-ups, participation in our research was offered to mothers. The cohort included children whose mothers agreed to participate and signed an informed consent form.

The first phase of research

The first phase of the research consisted of 127 children who had been evaluated at the age of 1, of which 92 had also been evaluated at the age of 2. The children were clinically examined, and the absolute eosinophil count; total IgE levels; specific IgE levels (inhalant and nutritive allergens); and RSV-specific IgG, IgG3, IgG4 and IgE antibodies were determined.

The second phase of research

The second phase of the research consisted of evaluating the children at the age of 10. A clinical examination was performed, along with the recording of anamnestic data and a review of medical documentation. The parents filled out a standardized International Study of Asthma and Allergies in Childhood (ISAAC) questionnaire, blood was sampled from a peripheral vein for planned laboratory analyses, and a skin prick test was performed. Through the anamnestic data, insights into the medical documentation, the clinical examination, and the standardized ISAAC questionnaire, we determined the incidence of atopic diseases (recurrent wheezing/asthma, allergic rhinitis and/or rhinoconjunctivitis, and atopic dermatitis) [35,36]. A total of 10 mL of blood was sampled from the peripheral vein, and serum for analyses that were not immediately performed was stored at −80 °C. The absolute number of eosinophils was determined from whole blood, while the total IgE, specific IgE for inhalant allergens, and vitamin D3 were determined from serum. The skin prick test was performed for inhalant allergens. Children performed spirometry after fractional exhaled nitric oxide (FeNO) measurement. Information about the occurrence of symptoms of recurrent wheezing/asthma, allergic rhinitis/rhinoconjunctivitis, and atopic dermatitis was collected by recording anamnestic data and analyzing medical documentation, performing a clinical examination, and administering a written standardized ISAAC questionnaire.

### 2.1. Absolute Eosinophil Count

The absolute eosinophil count in children at the age of 10 years was assessed with the hematological analyzer Sysmex XN-1000 (Sysmex Corporation, Kobe, Japan) [37].

### 2.2. Total IgE

The determination of the level of total IgE in children aged 10 years was analyzed using the electrochemiluminescence method (ECLIA-electrochemiluminescence immunoassay) on the immunochemical analyzer Cobas e601 (Roche Diagnostics, Tokyo, Japan) [38].

### 2.3. Allergen-Specific IgE

Specific IgE (*Dermatophagoides pteronyssinus*, *Dactylis glomerata*, *Ambrosia artemisiifolia*) was determined using a chemiluminescent enzyme immunoassay (CLEIA) with Allergen-Specific IgE 3g Allergy (Immulite, Siemens Healthcare GmbH, Erlangen, Germany) reagents on an IMMULITE^®^ 2000 Xpi analyzer (Siemens Healthcare GmbH, Erlangen, Germany) [39], and other specific IgE analyses (cat and dog dander, *Betula verrucosa*, *Corylus avellana*, *Artemisia vulgaris*, *Alternaria alternata*, *Cladosporium herbarum*) were carried out using a fluoroenzyme immunoassay (FEIA) with ImmunoCAP (Phadia AB, Thermo Fisher Scientific, Uppsala, Sweden) reagents on a Phadia 100 Laboratory system (Phadia AB, Thermo Fisher Scientific, Waltham, MA, USA) [39]. The results obtained using Immulite and ImunoCAP proved to be approximately sensitive and comparable, even though complete uniformity of results is not possible [39,40].

### 2.4. Prick Test

Skin testing for inhalant allergens (*Dermatophygoides pteronyssinus*, cat and dog dander, *Betula verrucosa*, *Corylus avellana*, *Dactylis glomerata*, *Ambrosia artemisiifolia*, *Artemisia vulgaris*) was carried out using the prick method [41]. We used allergen extracts from Diater Laboratorio De Diagnóstico Y Aplicaciones Terapeuticas SA, Madrid, Spain. A histamine solution (10 mg/mL) was used as a positive control, and normal saline was used as a negative test. A drop of allergen was applied to the skin of the volar side of the forearm at intervals of at least 2 cm. The skin was pricked with a standardized lancet (Stallerpoint) at an angle of 90°. After 20 min, the largest diameter of the wheal was measured. The largest diameter of the wheal greater than 3 mm or more from the negative control was evaluated as a positive skin test [42].

### 2.5. Additional Analyses

The level of 25-OH-vitamin D3 was analyzed using the high-performance liquid chromatography (HPLC) method with a commercial kit Eureka 25-OH-Vitamin D3 in plasma UV FAST (EUREKA LAB DIVISION S.r.l. (Chiaravalle, Ancona, Italy) on the 1260 Infinity Bio-Inert Quaternary HPLC device (Agilent Technologies, Santa Clara, CA, USA). Spirometry and FeNO were measured on a FeNO+ device (Medisoft, Sorinnes, Belgium).

The study was approved by the Medical Ethics Committee of the General County Hospital Požega, Croatia (chairperson: Tomislav Vuković, protocol number: 02-7/19-1/5-2009, date of approval: 17 April 2009); Faculty of Medicine, J. J. Strossmayer University of Osijek (chairperson: Ivan Požgain, protocol number: 2158-61-07-19-128, date of approval: 11 October 2019) and was conducted in accordance with the Helsinki Declaration. The patients were included in the study after obtaining written informed consent from their parents.

### 2.6. Statistical Analysis

Non-parametric statistics were used in the data analysis due to data distribution and the results of the Kolmogorov–Smirnov test. Continuous data are shown as medians and interquartile ranges, while categorical data are shown as frequencies and corresponding percentages. A Kaplan–Meier survival curve was used to analyze the group of children who had recurrent wheezing from the age of 2 to 10 with regard to the age of onset of the first wheezing episode. The Fisher–Freeman–Halton test was used for an analysis of the differences in the categorical variables. Kendal’s Tau_b correlation coefficients were used to analyze the correlations between the clinical findings (recurrent wheezing/asthma, atopic dermatitis, allergic rhinitis, and rhinoconjunctivitis) and RSV-specific antibodies. The multivariate prediction of positive specific IgE findings at the age of 10 in relation to RSV-specific antibodies and the multivariate predictions of the influence of risk factors on the occurrence of asthma and allergic rhinitis during the 10-year follow-up were analyzed using binary logistic regression. All *p* values below 0.05 were considered significant. All statistical procedures were carried out with IBM SPSS Statistics version 25.0.

## 3. Results

### 3.1. Descriptive Analysis of Study Population

The majority of patients—42 (58.33%)—were boys, while 44 (61.11%) of all included children had a positive family history of allergies. Twenty (27.78%) children were antenatally exposed to maternal smoking. A total of 53 (73.61%) of the children were delivered naturally (vaginally), with a median gestational age (interquartile range) of 274.0 (271.0–280.0) days. The median duration of exclusive breastfeeding was 3.0 (0.0–6.0) months, and that of total breastfeeding was 4.5 (2.0–12.8) months. Nine (12.68%) children attended nursery school. More than half of the patients lived with animals (inside or outside of the home) (Table S1).

### 3.2. Clinical and Laboratory Analyses of Study Population

By the age of two, 12 (16.7%) children had recurrent wheezing, while 22 (30.6%) children had recurrent wheezing from their 2nd to their 10th year of age. Atopic dermatitis was present in 18 (25.0%) subjects, allergic rhinitis was present in 32 (44.4%) subjects, and allergic rhinoconjunctivitis was present in 14 (19.4%) subjects followed up to 10 years of age. Symptoms in the last 12 months (current symptoms) were present in 15 (20.8%) children with wheezing, 7 (9.7%) children with atopic dermatitis, 29 (40.3%) children with allergic rhinitis, and 13 (18.1%) children with allergic rhinoconjunctivitis. A total of 20 (27.8%) children were monosensitized to inhalant allergens, and 24 (33.3%) were polysensitized, while 22 (30.6%) children were not sensitized to inhalant allergens at the age of 10. The median (interquartile range) concentration of total IgE was 75.13 (39.01–238.50) IU/mL, and the absolute eosinophil count was 200.0 (120.0–330.0) mcL (Table S2).

Children who had recurrent wheezing from 2 to 10 years of age were diagnosed with their first wheezing episode significantly earlier (χ2 8.097, df = 1, *p* = 0.004).

### 3.3. RSV-Specific Antibodies and Influence of Risk Factors on Atopic Diseases

The highest prevalence of RSV-specific antibodies was found among the IgG antibodies at the age of one year, with 109 (87.2%) in total, while at the age of two years, the total reached 82 (94.3%). Similar results were observed in the RSV-specific IgG3 antibodies during both years. The lowest prevalence of positive findings was among the RSV-specific IgE antibodies at the age of one year, with 18 (14.4%) in total, while at the age of two years, the total reached 6 (6.9%).

A positive correlation was found between the prevalence of asthma symptoms in the last 12 months in 10-year-old children and a higher concentration of RSV-specific IgG antibodies sampled at one year of age (tau_b = 0.196, *p* = 0.049).

Positive RSV-specific IgE antibodies the age of one increased the chances of allergic rhinitis occurrence by more than 15 times (OR = 15.03, 95% CI = 2.08–108.72; *p* = 0.007) when controlling for the influence of other risk factors in the regression model. Maternal smoking significantly increased the chances of allergic rhinitis occurrence during the follow-up by 7.63 times (OR = 7.63, 95% CI = 1.59–36.53; *p* = 0.011). The binary regression model for allergic rhinitis was statistically significant (*p* = 0.007), with 41.2% of explained dependent variable variance and 86% of correctly classified cases.

### 3.4. RSV-Specific Antibodies and Allergic Sensitization

There were no significant predictor variables in the prediction of positive allergen-specific IgE in the 10-year-old children in relation to the RSV-specific antibodies during the first two years of life.

## 4. Discussion

The results of the prospective cohort studies support the statement that early postnatal RSV infection is an independent risk factor for asthma development in children [12]. In the Avon Longitudinal Study of Parents and Children (ALSPAC), an association was found between RSV bronchiolitis in infancy and asthma in children aged seven years [12]. However, there are studies that do not advocate this premise [43]. A prospective study (the Tucson Respiratory Study) showed that RSV infection of the lower respiratory tract increased the risk of wheezing by age six, which persisted until age 11, but it was no longer detectable at age 13 [9]. Research in twins indicates that RSV increases the risk of wheezing in the short term, while the long-term effects are questionable [44].

### 4.1. Time of Acquisition of RSV Infection

**Figure 1:**
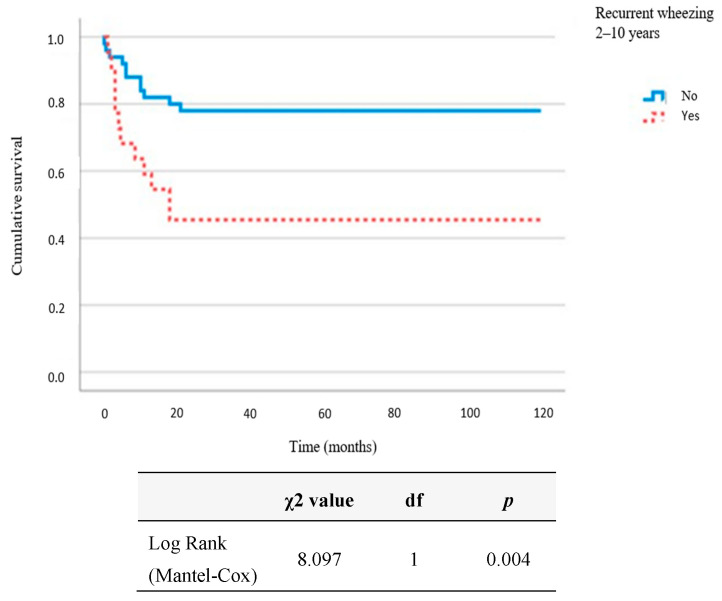
Kaplan–Meier curve considering age of onset of first wheezing episode and group of children who had recurrent wheezing from 2 to 10 years of age.

According to our results, the children with recurrent wheezing from the age of 2 to 10 years had their first wheezing episode at a significantly younger age (χ2 8.097, df = 1, *p* = 0.004) (Figure 1). Cohort studies have confirmed the first 18 months of life as a critical period for immune system modulation during respiratory infections [45]. Considering the age of acquisition of RSV infection, i.e., bronchiolitis, it has been stated that, the younger the child at the time of infection, the greater the risk of developing asthma in the future [44]. The age of children younger than six months at the time of their first RSV infection is associated with a higher prevalence of asthma in six-year-old children [44]. Recurrent respiratory infections may impair developing lungs and alter their function through the disruption of epithelial barrier integrity during childhood when the immune system is not fully mature. This could have a long-term impact on the lungs and the subsequent development of childhood asthma [46]. There are studies that link an older age at the time of infection to a higher risk of developing asthma in the future [20].

In the group of children with atopy, i.e., at least one positive immunoglobulin (Ig) E, 38.7% had been diagnosed with atopic dermatitis, while in the non-atopic group, 6 (14.6%) children had atopic dermatitis (*p* = 0.019).

### 4.2. RSV-Specific IgE Antibodies and Recurrent Wheezing/Asthma

In a group of children with atopy in our research, 35.5% had recurrent wheezing in comparison with 26.8% of non-atopic children from 2 to 10 years of age, but that difference was not significant (Table 1). In the RSV Bronchiolitis in Early Life (RBEL) study, children were followed after severe RSV bronchiolitis until the age of six, and it was found that the development of asthma and atopic dermatitis was not associated with Th2 phenotype indicators in peripheral blood (IgE and eosinophils) or with allergic sensitization [44]. Atopic and non-atopic wheezing are equally common in children aged 10 years, according to some studies [47]. Sigurs N. and colleagues found an association between severe RSV infection requiring hospitalization and allergic sensitization and asthma in children aged 7 and a half, 13, and 18 years [16,48,49].

The number of lower respiratory tract infections caused by RSV during the first years of life increases the risk of the subsequent development of asthma [13,14,15], and frequent RSV reinfections result in the accumulation of RSV-specific antibodies [5]. In an animal model, it was shown that viral infections can stimulate the atopic cycle via the induction of specific IgE antibodies (isotype switching) and lead to the development of allergic sensitization [29]. RSV can initiate an immediate hypersensitivity reaction via the induction of RSV-specific IgE antibodies, which can result in recurrent wheezing in childhood, as shown in a study in children following RSV bronchiolitis [30]. The results of the study conducted by Dakhama A. et al. documented the development of RSV-IgE responses in RSV-infected mice, and it established a role for RSV-specific IgE antibodies and the high-affinity IgE receptor in the development of exaggerated airway hypersensitivity reactions after an active RSV lung infection [50]. The described interactions might contribute to the development of airway dysfunction in children who develop RSV-IgE antibodies due to recurrent RSV infections. In our research, higher RSV-specific IgG antibodies sampled at the age of one year were positively correlated with current asthma symptoms in 10-year-old children (tau_b = 0.196, *p* = 0.049) (Table 2). This correlation had a weak level of biological association. Multivariate predictions of the influence of risk factors on asthma occurrence over the course of the 10-year follow-up showed that positive RSV-specific IgE antibodies sampled at the age of one increased the chances of asthma occurrence by 5.94 times (OR = 5.94, 95% CI = 1.05–33.64; *p* = 0.044) (Table 3).

A significant positive correlation was recorded between allergic rhinitis (tau_b = 0.290, *p* = 0.012) and allergic rhinitis in children with symptoms in the last 12 months (tau_b = 0.260, *p* = 0.025) and a higher concentration of RSV-specific IgE antibodies sampled at one year.

### 4.3. RSV-Specific IgE Antibodies and Allergic Rhinitis

We found that higher levels of RSV-specific IgE antibodies in the children sampled at the age of one were positively correlated with allergic rhinitis (tau_b = 0.290, *p* = 0.012) and current allergic rhinitis in 10-year-old children (tau_b = 0.260, *p* = 0.025) (Table 4). Multivariate predictions of the influence of risk factors on allergic rhinitis occurrence over the course of the 10-year follow-up showed that positive RSV-specific IgE antibodies at the age of one increased the chances of allergic rhinitis occurrence by more than 15 times (OR = 15.03, 95% CI = 2.08–108.72; *p* = 0.007) (Table 5). In the Oslo cohort study, in which children who had an early respiratory infection in the first year of life were followed, a higher rate of allergic sensitization and asthma was found, as well as an increased prevalence of allergic rhinitis at the age of 10 [51]. It has also been described that children with RSV infections in infancy have a higher prevalence of asthma and allergic rhinitis at the age of six [52].

### 4.4. RSV-Specific IgG4 Antibodies and Atopic Dermatitis

In children up to two years of age, the IgG subclasses found after primary RSV infection are mostly IgG1 and IgG3, which is a pattern typical of the response to viral proteins [53]. It is only in some children that RSV infection causes an increase in RSV-specific IgG4 and IgE antibodies [25]. A strong IgG response precedes or follows the appearance of IgE to the same molecule to which the organism is sensitized [54]. The simultaneous induction of RSV-specific IgG4 with RSV-specific IgE antibodies suggests that the IgG4-blocking antibodies could control IgE-induced allergic symptoms, possibly via mechanisms of the competitive inhibition of binding to viral peptides [26]. RSV-specific IgG3 antibodies have also been detected in the serum of children after an RSV infection [27]. RSV-specific IgG1 and IgG3 antibodies are predominant after a primary infection [55]. In our earlier research in children up to two years of age, a significant correlation was found between positive RSV IgG4 antibodies sampled at year one and atopic dermatitis (tau_b = 0.201, *p* = 0.025), as well as food allergy development (tau_b = 0.205, *p* = 0.023) until the second year of life [34]. According to our recent results, a higher concentration of RSV-specific IgG4 antibodies sampled in children at the age of one was borderline significantly positively correlated with atopic dermatitis (tau_b = 0.211, *p* = 0.049), and it was positively correlated with current atopic dermatitis in 10-year-old children (tau_b = 0.269, *p* = 0.012) (Table 6). RSV bronchitis during the first year of life is associated with the early development of atopic dermatitis, according to research by Singh A.M. et al. [56]. Similarly, children who had a moderate-to-severe respiratory infection (with or without wheezing) in the first 18 months of life have a higher chance of developing atopic dermatitis and allergic polysensitization in preschool age up to five years of age [57]. When viral pathogenesis has been investigated, RSV-associated wheezing in the first year of life has been found to be most closely associated with early/recurrent atopic dermatitis. This relationship suggests that these disorders are linked by a common underlying susceptibility factor, possibly related to immune regulation and epithelial barrier function [56].

### 4.5. RSV-Specific Antibodies and Allergic Sensitization

Furthermore, the result of our previous study showed that children who had a higher level of positive RSV-specific IgG4 antibodies at the age of one had a 2.73 times higher likelihood of having increased positive total and/or allergen-specific IgE antibodies during the first two years of life (OR = 2.73, 95% CI = 1.07–7.00; *p* = 0.036). The described probability is no longer present at the age of 10, according to our recent results (Table 7), which is consistent with the results of some studies [9]. This finding should be verified in a larger group of subjects. In the research of Sigurs N. et al., it was shown that, after recovering from RSV bronchiolitis, children have an increased risk of developing allergic sensitization and asthma in early adolescence [49]. A weak association with allergic sensitization in infants has been found in a large group of children with positive RSV-specific IgG antibodies [58]; opposite results were published in the Tucson study [9].

### 4.6. Risk Factors and Atopic Diseases

Atopic diseases have a heterogeneous etiology. It is believed that the onset of asthma depends on the interaction of at least two genetic, developmental, or environmental factors [59]. Evidence from animal models suggests the importance of viral infection as the initiating event that may trigger allergic airway inflammation, and it emphasizes the importance of the interaction between viral infection and exposure to inhaled allergens [60]. RSV bronchiolitis in infants increases the risk of asthma, and an allergic constitution (the presence of atopic dermatitis and elevated serum IgE) is an important prerequisite for the occurrence of asthma [61]. We found that a positive family history of atopy increased the chances of asthma occurrence during follow-up by 5.49 times (OR = 5.49, 95% CI = 1.01–30.07; *p* = 0.049); however, this result was borderline significant, and a longer duration of breastfeeding significantly lowered that chance (OR = 0.63, 95% CI = 0.45–0.89; *p* = 0.008), which is consistent with the results of previous studies (Table 3) [62]. Maternal smoking significantly increased the chances of allergic rhinitis occurrence during follow-up by 7.63 times (OR = 7.63, 95% CI = 1.59–36.53; *p* = 0.011), which has been confirmed by other authors (Table 5) [63].

Our study has some limitations. The first limitation is the relatively small number of participants for a cohort study. Our research cannot determine whether RSV infection by itself contributes to the increased frequency of atopic diseases or whether additional risk factors exist. Data from our study could be used to design larger confirmatory studies. The second limitation is the larger number of subjects with a positive family history of atopy in contrast to the general population and the unsolvable “chicken and the egg dilemma”. It remains unclear whether RSV infection in early childhood can be a cause of atopic diseases in the future or whether it only occurs in children with an allergic predisposition. The presence of RSV-specific IgG4 and IgE antibodies may only be markers for the atopic constitution present at the time of RSV infection.

Our research supports the fact that RSV infection can stimulate a specific immune response with the induction of RSV-specific IgG4 and IgE antibodies, and the presence of RSV-specific IgG4 and IgE antibodies can be a risk marker for the development of atopic diseases in children with atopy up to the age of 10 after RSV infection in infancy. The clinical implication of our study is the possibility of implementing prevention measures in children in whom an increase in RSV-specific IgE and IgG4 antibodies is recorded after RSV infection.

## 5. Conclusions

In our study, children with recurrent wheezing from 2 to 10 years of age had their first wheezing episode at a significantly younger age.

We reported significant correlations between higher levels of RSV-specific antibodies at age one and atopic diseases in children up to 10 years of age, i.e., between RSV-specific IgE antibodies and allergic rhinitis and between RSV-specific IgG4 antibodies and atopic dermatitis.

Positive RSV-specific IgE antibodies at the age of one increased the chances of asthma occurrence by 5.94 times, as well as the occurrence of allergic rhinitis by more than 15 times.

We also found that a positive family history increased the chances of asthma occurrence by 5.49 times, while a longer breastfeeding duration significantly lowered that chance. Maternal smoking during pregnancy significantly increased the chances of allergic rhinitis occurrence by 7.63 times.

### Clinical Consequences

The presence of RSV-specific IgE and IgG4 antibodies can be a risk marker for the development of atopic diseases in children with atopy up to the age of 10 after RSV infection in infancy.

## Figures and Tables

**Table 1:** Differences in frequency of recurrent wheezing, atopic dermatitis, allergic rhinitis, and rhinoconjunctivitis in children up to the age of 10 considering atopy (at least one positive specific IgE up to the age of 10): Fisher–Freeman–Halton test.

		≥1 Positive Specific IgE				*p*
		No		Yes		
		N	%	N	%	
Recurrent wheezing 2–10 years	No	30	73.2%	20	64.5%	0.430
	Yes	11	26.8%	11	35.5%	
Wheezing current	No	34	82.9%	23	74.2%	0.366
	Yes	7	17.1%	8	25.8%	
Atopic dermatitis <10 years	No	35	85.4%	19	61.3%	0.019
	Yes	6	14.6%	12	38.7%	
Atopic dermatitis current	No	39	95.1%	26	83.9%	0.111
	Yes	2	4.9%	5	16.1%	
Allergic rhinitis <10 years	No	24	58.5%	16	51.6%	0.558
	Yes	17	41.5%	15	48.4%	
Allergic rhinitis current	No	26	63.4%	17	54.8%	0.463
	Yes	15	36.6%	14	45.2%	
Allergic rhinoconjunctivitis <10 years	No	35	85.4%	23	74.2%	0.236
	Yes	6	14.6%	8	25.8%	
Allergic rhinoconjunctivitis current	No	35	85.4%	24	77.4%	0.385
	Yes	6	14.6%	7	22.6%	

**Table 2:** Correlation of wheezing in children until the age of 10 with RSV-specific antibodies sampled within the first 2 years of life (NovaTec-Units, NTU): Kendall’s tau_b correlation coefficient.

Respiratory Syncytial Virus (RSV)-Specific Antibodies		Recurrent Wheezing 2–10 Years	Wheezing Current	Recurrent Wheezing <2 Years
IgE 1st year	Correlation coefficient	0.219	0.174	0.093
	P	0.059	0.134	0.420
	N	70	70	70
IgG 1st year	Correlation coefficient	0.188	0.196	0.031
	P	0.059	0.049	0.755
	N	70	70	70
IgG3 1st year	Correlation coefficient	−0.082	0.008	−0.170
	P	0.412	0.936	0.088
	N	70	70	70
IgG4 1st year	Correlation coefficient	−0.105	−0.004	−0.065
	P	0.327	0.968	0.544
	N	69	69	69
IgE 2nd year	Correlation coefficient	0.111	0.039	0.050
	P	0.360	0.750	0.680
	N	67	67	67
IgG 2nd year	Correlation coefficient	−0.056	0.032	0.024
	P	0.578	0.756	0.813
	N	67	67	67
IgG3 2nd year	Correlation coefficient	0.010	−0.013	0.122
	P	0.922	0.896	0.229
	N	67	67	67
IgG4 2nd year	Correlation coefficient	−0.049	0.110	−0.119
	P	0.653	0.311	0.274
	N	67	67	67

A positive RSV-specific IgE antibodies at the age of one increased the chances of recurrent wheezing/asthma occurrence by 5.94 times (OR = 5.94, 95% CI = 1.05–33.64; *p* = 0.044) when controlling for the influence of other risk factors in the regression model. Moreover, positive family history increased the chances of recurrent wheezing/asthma occurrence during the follow-up by 5.49 times (OR = 5.49, 95% CI = 1.01–30.07; *p* = 0.049), while a longer duration of breastfeeding significantly lowered that chance (OR = 0.63, 95% CI = 0.45–0.89; *p* = 0.008). The binary regression model was statistically significant (*p* = 0.012), with 50.1% of an explained dependent variable variance and 85% of correctly classified cases.

**Table 3:** Multivariate prediction of the influence of risk factors on recurrent wheezing/asthma occurrence over the course of 10-year follow-up: binary logistic regression.

Recurrent Wheezing (2–10 Years), r^2^ = 50.1%, *p* = 0.012	OR	95% CI		*p*
		Lower	Upper	
Birth weight (g)	1.00	1.00	1.00	0.227
Positive family history	5.49	1.01	30.07	0.049
Maternal smoking during pregnancy	0.35	0.06	1.92	0.226
Male gender	2.08	0.47	9.18	0.334
Mode of delivery: caesarean section (CS)	3.42	0.73	16.08	0.119
Duration of exclusive breastfeeding (months)	0.63	0.45	0.89	0.008
Vitamin D (mcg/L)	1.02	0.91	1.14	0.762
Positive RSV-specific IgE—1 year	5.94	1.05	33.64	0.044
Positive RSV-specific IgG—1 year	0.34	0.04	2.97	0.326
Positive RSV-specific IgE—2 years	1.53	0.15	15.33	0.716
Positive RSV-specific IgG—2 years	0.10	0.01	1.03	0.053

**Table 4:** Correlation of allergic rhinitis and rhinoconjunctivitis in children up to the age of 10 and RSV-specific antibodies sampled within the first 2 years of life (NTU): Kendall’s tau_b correlation coefficient.

RSV-Specific Antibodies		Allergic Rhinitis <10 Years	Allergic Rhinitis Current	Allergic Rhinoconjunctivitis <10 Years	Allergic Rhinoconjunctivitis Current
IgE 1st year	Correlation coefficient	0.290	0.260	0.089	0.109
	P	0.012	0.025	0.441	0.349
	N	70	70	70	70
IgG 1st year	Correlation coefficient	−0.036	−0.016	−0.052	−0.014
	P	0.722	0.870	0.602	0.888
	N	70	70	70	70
IgG3 1st year	Correlation coefficient	0.010	−0.018	−0.057	−0.025
	P	0.920	0.856	0.566	0.803
	N	70	70	70	70
IgG4 1st year	Correlation coefficient	0.078	0.015	−0.044	−0.014
	P	0.468	0.885	0.683	0.899
	N	69	69	69	69
IgE 2nd year	Correlation coefficient	0.154	0.179	0.208	0.226
	P	0.206	0.141	0.087	0.064
	N	67	67	67	67
IgG 2nd year	Correlation coefficient	−0.071	−0.076	−0.035	−0.069
	P	0.486	0.455	0.731	0.498
	N	67	67	67	67
IgG3 2nd year	Correlation coefficient	−0.104	−0.078	−0.082	−0.031
	P	0.308	0.440	0.418	0.761
	N	67	67	67	67
IgG4 2nd year	Correlation coefficient	−0.003	−0.030	−0.060	−0.028
	P	0.979	0.780	0.579	0.795
	N	67	67	67	67

**Table 5:** Multivariate prediction of the influence of risk factors on allergic rhinitis occurrence over the course of 10-year follow-up: binary logistic regression.

Allergic Rhinitis (<10 Years), r^2^ = 41.2%, *p* = 0.007	OR	95% CI		*p*
		Lower	Upper	
Birth weight (g)	1.00	1.00	1.00	0.262
Positive family history	4.02	0.99	16.40	0.052
Maternal smoking during pregnancy	7.63	1.59	36.53	0.011
Male gender	1.52	0.41	5.63	0.527
Mode of delivery: caesarean section (CS)	1.11	0.29	4.27	0.885
Duration of exclusive breastfeeding (months)	0.86	0.67	1.11	0.251
Vitamin D (mcg/L)	1.01	0.91	1.12	0.842
Positive RSV-specific IgE—1 year	15.03	2.08	108.72	0.007
Positive RSV-specific IgG—1 year	0.30	0.05	1.87	0.198
Positive RSV-specific IgE—2 years	6.25	0.40	98.40	0.193
Positive RSV-specific IgG—2 years	0.28	0.04	1.80	0.180

A positive correlation was found between atopic dermatitis in children until the age of 10 years (tau_b = 0.211, *p* = 0.049) and atopic dermatitis with symptoms present in the last 12 months (tau_b = 0.269, *p* = 0.012), with a higher concentration of RSV-specific IgG4 antibodies sampled in children at the age of one year.

**Table 6:** Correlation of atopic dermatitis in children up to 10 years of age and RSV-specific antibodies sampled within the first 2 years of life (NTU): Kendall’s tau_b correlation coefficient.

RSV-Specific Antibodies		Atopic Dermatitis <10 Years	Atopic Dermatitis Current
IgE 1st year	Correlation coefficient	−0.085	−0.019
	P	0.461	0.870
	N	70	70
IgG 1st year	Correlation coefficient	0.090	0.054
	P	0.367	0.590
	N	70	70
IgG3 1st year	Correlation coefficient	0.009	0.117
	P	0.930	0.240
	N	70	70
IgG4 1st year	Correlation coefficient	0.211	0.269
	P	0.049	0.012
	N	69	69
IgE 2nd year	Correlation coefficient	−0.145	−0.085
	P	0.233	0.485
	N	67	67
IgG 2nd year	Correlation coefficient	0.037	0.133
	P	0.719	0.189
	N	67	67
IgG3 2nd year	Correlation coefficient	−0.018	0.086
	P	0.857	0.395
	N	67	67
IgG4 2nd year	Correlation coefficient	0.075	0.172
	P	0.492	0.113
	N	67	67

A significant positive correlation was recorded between allergic rhinitis (tau_b = 0.290, *p* = 0.012) and allergic rhinitis in children with symptoms in the last 12 months (tau_b = 0.260, *p* = 0.025) and a higher concentration of RSV-specific IgE antibodies sampled at one year.

**Table 7:** Prediction of positive allergen-specific IgE in 10-year-old children in relation to RSV-specific antibodies during the first 2 years of life: binary logistic regression.

RSV-Specific Antibodies	OR	95% CI		*p*
		Lower	Upper	
IgE 1st year	0.34	0.00	85.47	0.699
IgG 1st year	1.00	0.97	1.03	0.848
IgG3 1st year	0.41	0.16	1.09	0.073
IgG4 1st year	0.61	0.16	2.26	0.459
IgE 2nd year	0.00	0.00	6.35	0.137
IgG 2nd year	1.01	0.97	1.04	0.636
IgG3 2nd year	1.07	0.28	4.03	0.926
IgG4 2nd year	1.91	0.52	6.94	0.327

## Data Availability

The data associated with the paper are not publicly available but are available from the corresponding author on reasonable request.

## Supplementary Materials

The following supporting information can be downloaded at: https://www.mdpi.com/article/10.3390/pathogens12040546/s1, Table S1: Socio-demographic characteristics of included children up to the age of 10 (N = 72). Table S2: Clinical characteristics of included children up to the age of 10 (N = 72).
