# Supplementary material for: Respiratory Syncytial Virus-Specific Antibodies and Atopic Diseases in Children: A 10-Year Follow-Up
Source: Pathogens. 2023 Apr 1;12(4):546. doi: 10.3390/pathogens12040546 (PMC10142345; doi:10.3390/pathogens12040546)
Supplement: Supplementary file 1 [file pathogens-12-00546-s001.zip › pathogens-2218646-supplementary.pdf]

Supplementary Materials:

Table S1. Socio-demographic characteristics of included children up to the age of 10 (N = 72).

		N (%)	Median (25th, 75th)
Gender	Female	30 (41.67 %)	
	Male	42 (58.33 %)	
Family history	Negative	28 (38.89 %)	
	Positive	44 (61.11 %)	
Gestational age (days)			274.0 (271.0–280.0)
Mode of delivery	Vaginal	53 (73.61 %)	
	Caesarean section (CS)	19 (26.39 %)	
Birth weight (g)			3405.0(3150.0–3680.0)
Birth length (cm)			50.0 (48.5–50.0)
Head circumference (cm)			34.5 (33.5–35.0)
Neonatal pathology	Without	43 (59.72 %)	
	Hyperbilirubinemia	13 (18.06 %)	
	Hyperbilirubinemia and neonatal infection	12 (16.67 %)	
	Asphyxia and neonatal infection	2 (2.78 %)	
	Apnea and pneumothorax and neonatal infection	1 (1.39 %)	
	Convulsions	1 (1.39 %)	
Animals (1st year)	No	30 (42.25 %)	
	Yes	41 (57.75 %)	
Animals (2nd year)	No	32 (45.07 %)	
	Yes	39 (54.93 %)	
Number of adults in the household (1st year)			2.0 (2.0–2.0)
Number of children in the household (1st year)			2.0 (1.0–2.0)
Number of adults in the household (2nd year)			2.0 (2.0–2.0)
Number of children in the household (2nd year)			2.0 (1.0–3.0)
Nursery	No	62 (87.32 %)	
	Yes	9 (12.68 %)	
Maternal smoking during pregnancy	No	52 (72.22 %)	
	Yes	20 (27.78 %)	
Household smoking	No	43 (59.72 %)	
	Yes	29 (40.28 %)	
Exclusive breastfeeding (months)			3.0 (0.0–6.0)
Total breastfeeding (months)			4.5 (2.0–12.8)

Continuous variables are presented as medians and 25th and 75th percentiles, and categorical variables are presented as frequencies (n) and percentages (%).

Table S2. Clinical characteristics of included children up to the age of 10 (N = 72).

		N (%)	Median (25th, 75th)
Recurrent wheezing 2–10 years	No	50 (69.4 %)	
	Yes	22 (30.6 %)	
Wheezing current	No	57 (79.2 %)	
	Yes	15 (20.8 %)	
Recurrent wheezing < 2 years	No	60 (83.3 %)	
	Yes	12 (16.7 %)	

		N (%)	Median (25th, 75th)
Atopic dermatitis < 10 years	No	54 (75.0 %)	
	Yes	18 (25.0 %)	
Atopic dermatitis current	No	65 (90.3 %)	
	Yes	7 (9.7 %)	
Allergic rhinitis < 10 years	No	40 (55.6 %)	
	Yes	32 (44.4 %)	
Allergic rhinitis current	No	43 (59.7 %)	
	Yes	29 (40.3 %)	
Allergic rhinoconjunctivitis <10 years	No	58 (80.6 %)	
	Yes	14 (19.4 %)	
Allergic rhinoconjunctivitis current	No	59 (81.9 %)	
	Yes	13 (18.1 %)	
Groups	Elevated total IgE	6 (8.3 %)	
	Monosensitization	20 (27.8 %)	
	Polysensitization	24 (33.3 %)	
	Negative total and specific IgE	22 (30.6 %)	
Absolute eosinophil count >300/mcL	No	50 (69.4 %)	
	Yes	22 (30.6 %)	
FeNO (ppb)			8 (6–13)
Vitamin D (mcg/mL)			21 (16–25)
Absolute eosinophil count (mcL)			200.00 (120.00–330.00)
IgE (IU/mL)			75.13 (39.01–238.50)

Continuous variables are presented as medians and 25th and 75th percentiles, and categorical variables are presented as frequencies (n) and percentages (%).
